# P3/P3N-PIPO of PVY interacting with BI-1 inhibits the degradation of NIb by ATG6 to facilitate virus replication in *N. benthamiana*

**DOI:** 10.3389/fpls.2023.1183144

**Published:** 2023-04-17

**Authors:** Zhen Qing, Shakeel Ahmad, Yuemeng Chen, Qingmin Liang, Lijuan Zhang, Baoshan Chen, Ronghui Wen

**Affiliations:** ^1^ State Key Laboratory for Conservation and Utilization of Subtropical Agro-bioresources, College of Life Science and Technology, Guangxi University, Nanning, China; ^2^ College of Agriculture, Guangxi University, Nanning, China

**Keywords:** Autophagy, Beclin1 (ATG6), NbBI-1, *N. benthamiana*, RNA-dependent RNA polymerase (RdRp)

## Abstract

**Introduction:**

Autophagy not only plays an antiviral role but also can be utilized by viruses to facilitate virus infection. However, the underlying mechanism of potato virus Y (PVY) infection against plant autophagy remains unclear. BI-1, localizing to the endoplasmic reticulum (ER), is a multifunctional protein and may affect the virus infection.

**Methods:**

In this study, Y2H, BiFC, qRT-PCR, RNA-Seq, WB and so on were used for research.

**Results:**

P3 and P3N-PIPO of PVY can interact with the Bax inhibitor 1 (BI-1) of *N. benthamiana*. However, BI-1 knockout mutant showed better growth and development ability. In addition, when the BI-1 gene was knocked out or knocked down in *N. benthamiana*, the PVY-infected mutant showed milder symptoms and lower virus accumulation. Analysis of transcriptome data showed that the deletion of NbBI-1 weakened the gene expression regulation induced by PVY infection and NbBI-1 may reduce the mRNA level of NbATG6 by regulated IRE1-dependent decay (RIDD) in PVY-infected *N. benthamiana*. The expression level of the ATG6 gene of PVY-infected WT was significantly down-regulated, relative to the PVY-infected mutant. Further results showed that ATG6 of *N. benthamiana* can degrade NIb, the RNA-dependent RNA polymerase (RdRp) of PVY. NbATG6 has a higher mRNA level in PVY-infected BI-1 knockout mutants than in PVY-infected WT.

**Conclussion:**

The interaction of P3 and/or P3N-PIPO of PVY with BI-1 decrease the expression of the ATG6 gene might be mediated by RIDD, which inhibits the degradation of viral NIb and enhances viral replication.

## 1 Introduction

BI-1, a highly conserved cell death suppressor, is first identified based on its suppression of Bax-activated cell death in yeast (Xu and Reed, 1998). BI-1 localizes to the endoplasmic reticulum (ER) membrane and has an important cytoprotective function in responses to biotic and abiotic stresses (Watanabe and Lam, 2009). BI-1 has been implicated in several human diseases, including chronic liver disease, diabetes, ischemia/reperfusion injury, neurodegeneration, and cancer (Lebeaupin et al., 2020). In plants, BI-1 controls the response to environmental signals that cause cell death by activating the conserved ER stress response pathway (Watanabe and Lam, 2009). However, BI-1 shows dual regulatory roles of resistance or susceptibility in the different plant pathosystems. Transient expression of barley BI-1 weakens oxidative defense and resistance to the biotrophic fungal pathogen *Blumeria graminis* (Eichmann et al., 2006). Similarly, silencing of barley BI-1 increases resistance to powdery mildew, suggesting BI-1 is a susceptibility factor toward powdery mildew (Eichmann et al., 2010). Conversely, the expression of barley BI-1 in carrots confers resistance to Botrytis cinerea and Chalara elegans (Imani et al., 2006). These findings imply that BI-1 may function as a flexible molecular switch in plants, responding to a range of biotic or abiotic stimulus signals (Watanabe and Lam, 2009). However, the molecular mechanism of BI-1 regulatory roles in resistance or susceptibility is still unknown when the host is infected with a pathogen.

During the interaction between pathogen and host, both BI-1 and autophagy show the potential of bidirectional regulation, prosurvival or prodeath. BI-1 may act as a calcium channel and contribute to the homeostasis of calcium in the endoplasmic reticulum (Sano et al., 2012; Chang et al., 2014; Lebeaupin et al., 2020). In animal cells, BI-1 has been shown to promote autophagy by regulating lysosomal calcium (Kim et al., 2021), and influencing mitochondrial bioenergetics, reducing oxygen consumption, impacting cellular ATP levels, and stimulating autophagy (Sano et al., 2012). In plants, only one report shows that BI-1 interacts with ATG6 of *N. benthamiana*, and silencing of BI-1 reduced the autophagic activity induced by both N gene-mediated resistance to tobacco mosaic virus and methyl viologen (Xu et al., 2017). The specific linkage mechanism between the BI-1 and autophagy is hitherto largely unclear. The role of BI-1 in autophagy deserves further exploration.

Autophagy is an evolutionary conserved intracellular self-cleaning and renewal system, by which cytoplasmic proteins and dysfunctional organelles, are delivered to lysosomes or vacuoles for degradation and recycling (Liu and Bassham, 2012; Yang and Bassham, 2015). Autophagy is grouped into three main forms in eukaryotic cells: macroautophagy, microautophagy, and chaperone-mediated autophagy (Klionsky, 2005; Mizushima, 2007). Through extensive genetic mutation, 40 genes are found to be autophagy-related genes (*ATGs*) in yeast (Ke, 2017).

Recently, Autophagy induced by plant virus infection has been widely reviewed (Kushwaha et al., 2019; Huang et al., 2020; Ismayil et al., 2020; Yang and Liu, 2022). Autophagy is reported to participate in the antiviral reaction. Tomato ATG8f interacts with the TrAP of Tomato leaf curl New Delhi virus to mediate TrAP degradation by the autophagy pathway, providing defense against viral infection (Prasad and Prasad, 2022). In *N. benthamiana*, Silencing of NbeIF4A activates autophagy and inhibits infection of rice stripe virus (RSV) by facilitating autophagic degradation of p3 of RSV (Zhang et al., 2021). NbATG6 (Beclin1), a core component of autophagy interacts with TuMV NIb resulting in its degradation and restricting virus infection (Li et al., 2018).

In turn, many viruses have evolved tactics to antagonizing autophagy that exploit host autophagy mechanisms for viral replication, or promote host fitness for viral infection. During infection, some defense-related host factors are reported to be targeted by pathogens for autophagic degradation, thus enhancing pathogen virulence. The NSs of tomato zonate spot virus reduce the concentration of NbSGS3 protein through the autophagy pathways resulting in the attenuation of antiviral RNA silencing (Chen et al., 2022). Interaction between P0 of Brassica yellows virus silencing suppressor and SKP1 of *N.benthamiana* suppress autophagic degradation of P0 to enhance system infection (Li et al., 2019). Moreover, bamboo mosaic virus infection induces the expression of ATGs to assist the virus replication in *N.benthamian* (Huang et al., 2019). The long-term survival of viruses in plant cells produces a range of interactions, Co-adaptation balances the fitness between the host and virus. However, it is still poorly understood how viruses respond to autophagy to promote their replication.

PVY is a widely distributed virus worldwide, which causes huge economic losses to many major crops (Li et al., 2022a). PVY can encode multiple viral proteins, including P3, P3N-PIPO, and NIb. P3 protein locates at ER which is essential for the functionality of P3 protein (Eiamtanasate et al., 2007). Plant potyviruses assemble viral replication complexes (VRCs) on modified cellular membranes as other positive-strand RNA viruses and the C-terminal region of TuMV P3 is indispensable for P3 to form cytoplasmic punctate inclusions and target VRCs (Cui et al., 2017). P3 is also involved in TuMV-induced symptoms (Kim et al., 2019). As a virulence determinant in plants resistant to a potyvirus, P3N-PIPO is involved in overcoming cyv1-mediated resistance (Choi et al., 2013). WYMV (Wheat Yellow Mosaic Virus) P3N-PIPO interacts with host protein NbRLK6, and silencing the expression of the NbRLK6 gene reduces the severity of disease and the level of virus accumulation caused by PVX-P3N-PIPO infection in *N.benthamiana* (Miao et al., 2022). TuMV P3N-PIPO interacts with P3 via the shared P3N domain for cell-to-cell movement (Chai et al., 2020). Potato Y virus NIb protein is an RNA-dependent RNA polymerase, mainly responsible for the replication of the virus genome (Shen et al., 2020). The interaction between TVBMV NIb and NbRPL1 in chloroplast reduces the degradation of NIb by NbBeclin1 and enhances virus infection (Cheng et al., 2021).

PVY has many strains, having different phenotypic characters and resistance patterns, which are mainly host-dependent (Whitworth et al., 2021). But there is still a lack of a complete mechanistic understanding of the roles of different autophagy-related host factors in controlling virus infection. We assumed that there is a correlation between these phenotypes and BI-1 mediated autophagy pathway. Our results revealed that during PVY infection, PVY P3 and P3N-PIPO interacted with BI-1 of *N benthamiana*. Furthermore, the infection of PVY resulted in negative regulation of ATG6 mRNA, which subsequently inhibited the degradation of NIb proteins. Our results suggest that P3/P3N-PIPO of PVY interacting with BI-1 promotes virus replication by inhibiting the degradation of viral protein NIb.

## 2 Materials and methods

### 2.1 Virus strain, plant material, and chlorophyll content detection

The PVY isolate identified in this study was the PVY^N-Wi^ strain group. The *N. benthamiana* seeds used in this study were supplied by Suzhou Yikai Co., Ltd. (Suzhou, China). Plants were grown in special potting soil (2 parts matrix soil: 1 part Vermiculite) in a growth chamber at 24°C ~ 26°C with a 16 h photoperiod and at 75% RH (Relative Humidity). After the plant seeds were soaked overnight in distilled water at room temperature and then planted, the seed germination rate was measured on the 14^th^ day after the plant seeds were cultured. The growth of seedlings was observed daily after sowing, and photos were taken for record. Furthermore, leaves were collected for chlorophyll content detection (Kim and Kim, 2013). Select 2-week-old and 4-week-old *N. benthamiana* and WT as the control group. After stopping watering for 10 days, the differences between WT, KD, and KO were recorded and compared (Zhang et al., 2017). Select 4-week-old *N. benthamiana* and WT as the control group. After culturing at 10 °C for 2 months, the differences in growth of WT, KD, and KO were recorded and compared (Shi et al., 2014).

### 2.2 BiFC and Y2H assays

P3, P3N-PIPO, and NbBI-1 genes were combined with the BiFC destination vectors (C-YFP-pMBIA1301, N-YFP-pMBIA1301) to generate the N-terminal YFP or C-terminal YFP fusion constructs. The plasmids of N-YFP-P3, N-YFP-P3N-PIPO, and C-YFP-NbBI-1 were constructed and transformed into *Agrobacterium tumefaciens*. The transformed *Agrobacteriums*, including N-YFP-P3 or N-YFP-P3N-PIPO and C-YFP-NbBI-1, were co-infiltrated in *N. benthamiana* epidermal leaves by 1 mL syringes. BiFC signal was analyzed using a confocal laser microscope (LEICA-TCS-SP8MP; Germany), with a 488 nm argon laser for YFP excitation, and a 530 ~ 550 nm filter was used for Emission signals to be detected.

The Matchmaker Gold Yeast Two-Hybrid System (Clontech, DUAL membrane Starter Kit) was used for Y2H assays to assess the interaction between PVY proteins and NbBI-1. Genes encoding PVY mature protein were inserted into pDHB1 bait plasmids, NbBI-1 gene was inserted into the pPR3-N prey plasmid. Plasmids pDHB1-P1, pDHB1-HC-Pro, pDHB1-P3, pDHB1-P3N-PIPO, pDHB1-6K1, pDHB1-CI, pDHB1-6K2, pDHB1-NIa, pDHB1-NIb, pDHB1-VPg and pDHB1-CP were constructed. They were transferred into yeast NMY51 (G6041, ANYUBIO, Shanghai, China) and cultured on the defective medium.

### 2.3 *Agrobacterium*−mediated transient expression in *N. benthamiana* and virus infection

The expression vector was constructed in the binary vector pCAMBIA3300, 2×35S CaMV, target gene (*NbATG6* and *NIb*), NOS elements, protein labels Myc and Flag, were added to the MCS region. The constructs were verified through sequencing and chemically converted into the *A. tumefaciens* strain EHA105. Recombinant strains of *A. tumefaciens* were cultivated in YEP medium with 100 µg/ml kanamycin and 25 µg/ml rifampicin at 28°C at 200 rpm shaking for 48 hours to an OD600 of 0.8 ~ 1.2. The cultures were harvested, washed with Agrobacterium inoculum buffer (0.15M AS 50 uL, 0.1M MES 5 mL, 0.1M MgCl_2_ 5 mL, H_2_O 39.95 mL) two times and resuspended in Agrobacterium inoculum buffer to a final OD600 of 0.5 ~ 0.8. The *A. tumefaciens* cell suspension was infiltrated into *N. benthamiana* leaves using a syringe without a needle (Xie et al., 2019).

Enough virus-infected leaves were weighed and grounded with liquid nitrogen, then mixed with 2 times the volume of viral inoculation buffer (K_2_HPO_4_ 1 g, Na_2_SO_3_ 0.1 g, H_2_O 100 mL). The supernatant was obtained after centrifugation (12000 rpm/min, 10 mins) to inoculate the *N. benthamiana* leaves by friction (Each plant was inoculated with 100 uL supernatant, one leaf per 50 uL) (Zhang et al., 2022).

### 2.4 RNA isolation and qRT-PCR assays

The HiPure Plant RNA Mini Kit (Magen, Shanghai, China) was used to isolate total RNA from frozen leaves of *N. benthamiana* (1^st^ or 2^nd^ leaf). Subsequently, cDNA was synthesized using TransScript^®^ One-Step gDNA Removal and cDNA Synthesis SuperMix (TransGen-Biotech, Beijing, China). All qPCR experiments were performed using TransStart Green qPCR SuperMix (TransGen-Biotech, Beijing, China). **Table S1** shows the oligonucleotide primer set used for qPCR. The normalized treatment was performed with the GAPDH gene of *N. benthamiana* as a reference. For each gene measurement, 3 to 5 biological replicates were used in each treatment. qPCR data analysis and primer efficiency were obtained using the EXCEL software, and PRISM was used to construct figures. Relative expression rate and statistical analysis the 2^-ΔΔCt^ method was used to analyze the relative changes in gene expression (Livak and Schmittgen, 2001).

### 2.5 ELISA and western blot assays

Plant tissue (3^rd^ or 4^th^ leaf) was grinded in Liquid nitrogen, and the PVY Reagent Set (Agdia, Elkhart, USA) was used to test the results of PVY accumulation. After adding the substrate for color development, the value of OD_480_ was recorded every 10 minutes. The relative content of PVY was calculated by the change in OD_480_ values. Data analysis performed by EXCEL software and PRISM was used to draw figures.

Total leaf proteins were extracted with a plant total protein extraction kit (BestBio, Shanghai, BB-3124). Protein extraction samples were analyzed by SDS-PAGE, immunoblotted using anti-Flag (Beyptime, AF519), anti-Myc (Beyptime, AM926), anti-GFP (Beyptime, AG281) and anti-Rubisco (Abbkine, ABM40218) anti-bodies. Protein extraction samples were detected using a DAB colour developing kit (brown-yellow) western blotting substrate (BOSTER, AR1021). For quantification of DAB staining intensity, images were converted to grayscale and inverted, and then the mean gray of protein bands was calculated using Adobe Photoshop 2020.

### 2.6 Transcriptome analysis

At least 12 leaves from WT (Treat, 14 dpi), WT (Control, virus-free), KO (Treat, 14 dpi), and KO (Control, virus-free) plants were collected for RNA-Seq analysis. Total RNA was treated with the mRNA enrichment method (magnetic beads with OligodT were used to enrich mRNA with polyA tail) and rRNA removal method (Hybridization of rRNA with DNA probe, selective digestion of DNA/RNA hybridization chain by RNaseH, digestion of DNA probe by DNaseI, purification of required RNA). The obtained RNA was segmented by interrupting buffer, reverse-transcribed by random N6 primers, and then synthesized cDNA double strand to form double-stranded DNA. The synthetic double-stranded DNA ends are patched flat and phosphorylated at the 5’ end, forming A sticky end with an ‘A’ protruding at the 3’ end, then connected to a bubbling connector with a ‘T’ protruding at the 3’ end. The linked products were amplified by PCR using specific primers. The PCR product was thermal-denatured into a single strand. Then a bridge primer was used and classify the single-strand DNA to obtain a single-strand circular DNA library. The libraries were sequenced using the DNBSEQ sequencing platform according to the manufacturer’s instructions (BGI, China). The polished transcripts of each sample were mapped to the *N. benthamiana* reference genome (Nbv5.1_sefapps02.qut.edu.au, http://benthgenome.qut.edu.au/) using GMAP. The DEGs were identified and analyzed from RNA-Seq data with the Website (https://report.bgi.com/ps/login/login.html) of BGI. The database used in the analyses included GO (http://www.geneontology.org/), KEGG (http://www.genome.jp/kegg/), and NR (ftp://ftp.ncbi.nlm.nih.gov/blast/db/).

### 2.7 Statistical analysis

The data were analyzed with an Excel 2019 (Microsoft Inc., Redmond, USA) using a two-tailed Student’s t-test. All the data were expressed as standard error mean ( ± s.e.m) at P < 0.01 or P < 0.05. The graphs were constructed using GraphPad Prism 8.0 software.

## 3 Results

### 3.1 PVY P3 and P3N-PIPO interact with NbBI-1

**Figure 1:**
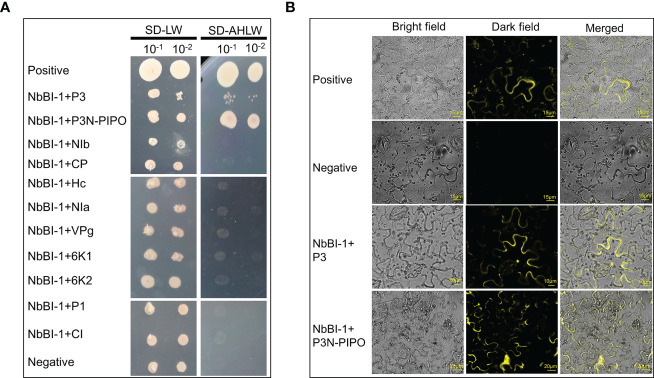
Interaction of NbBI-1 with PVY-encoded proteins **(A)** NbBI-1 interaction with PVY-encoded P3N-PIPO and P3 by Y2H. Negative: pTSU2-APP+pPR3-N. Positive: pTSu2-APP+pNubG-Fe65. **(B)** NbBI-1 interactions with P3 and P3N-PIPO by BiFC. Negative: N-YFP+C-YFP. Positive: N-YFP-TuMV-P3N-PIPO+C-YFP-AtPcaP1.

Initial studies were conducted to identify host proteins interacting with the P3N-PIPO of PVY. A cDNA library of *N. benthamiana* was used for Y2H assays with P3N-PIPO as the bait. One of the candidate proteins designated as NbBI-1 in *N. benthamiana*, which potentially showed strong interaction, was selected for further analysis (**Figure 1A**). The cDNA of the NbBI-1 gene was subsequently cloned and analyzed. To further validate this interaction, performing BiFC assays in the leaves of *N. benthamiana*. YFP fluorescence was observed in N-YFP-P3N-PIPO/C-YFP-NbBI-1 and positive combinations (**Figure 1B**). Since P3 and P3N-PIPO have the same N-end (Wen et al., 2011; Chai et al., 2020), The interaction between P3 of PVY and NbBI-1 was further confirmed by Y2H and BiFC assay (**Figure 1**). Similarly, the Y2H experiment was done to co-express NbBI-1 with other viral mature proteins of PVY. None of these proteins was found to interact with NbBI-1 in yeast (**Figure 1A**). These results suggest that NbBI-1 only interacts with the P3/P3N-PIPO of PVY-coded proteins.

### 3.2 NbBI-1 plays a negative role in the growth and development of *N. benthamiana*

**Figure 2:**
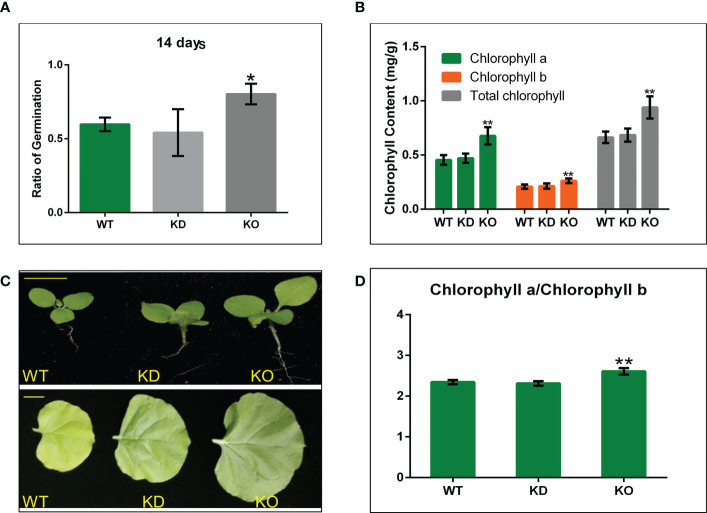
NbBI-1 regulatory role in the growth and development of *N. benthamiana* and NbBI-1 knockout reduce resistance to abiotic stresses. **(A)** Shows seeds germination rate after 14 days of sowing. **(B)** Growth and development *N. benthamiana*. Row 1, seedling size (14 days after sowing. Bar, 1cm), and Row 2 (record after 4 weeks of seeding. Bar, 2cm). **(C, D)** NbBI-1 inhibit chlorophyll contentin *N. benthamiana*. Chlorophyll a, chlorophyll b, Total chlorophyll, and the chlorophyll a/b ratio; in *N. benthamiana* leaves. Data are mean ± SEM, asterisks indicate a significant difference (Student’s t-test, test, *: 0.01<P<0.05, **: P<0.01).

To better investigate the function of NbBI-1 during PVY infection of *N. benthamiana*, we down-regulated the expression of the NbBI-1 by RNAi technique and deleted NbBI-1 through gene editing technology CRISPR/Cas9 to obtain NbBI-1 mutants, which were named KD mutant (**Figure S1**) and KO mutant (**Figure S2**). Although BI-1 knockout in Arabidopsis had no significant effect on growth (Watanabe and Lam, 2006), BI-1 knockout in fungi U. virens promoted mycelial growth and conidiation (Xie et al., 2019). Thus, after obtaining the NbBI-1 mutants, we first tested the normal growth and development of WT and NbBI-1 mutants. The results showed that the germination rate of KO was higher than that of KD and WT. However, no significant difference was observed between the KD and WT (**Figure 2A**). Moreover, the KO mutant showed faster growth and development than the WT and KD mutant (**Figure 2B**). The increasing chlorophyll content and chlorophyll a/b ratio benefit photosynthesis under higher light intensity (He et al., 2021). As a result, we also determined the leaves’ chlorophyll concentration. We noted that KO leaves had much higher levels of total chlorophyll, chlorophyll a, and b than WT and KD leaves, whereas WT and KD leaves were similar (**Figure 2C**). Moreover, KO had a considerably greater chlorophyll a/b ratio than WT and KD (**Figure 2D**). All above, NbBI-1 plays a negative role in the growth and development of *N. benthamiana*.

### 3.3 NbBI-1 Knockout suppresses PVY infection, and reduces resistance to abiotic stresses

**Figure 3:**
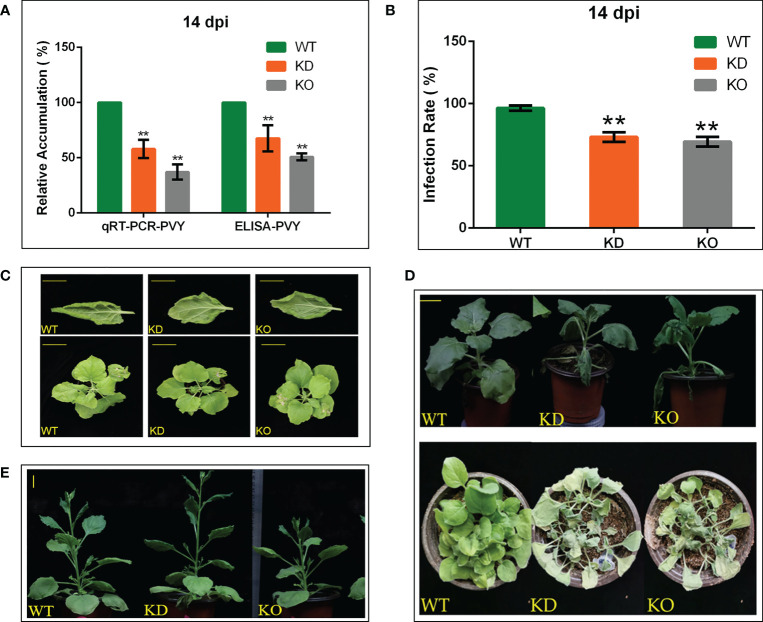
NbBI-1 promotes PVY accumulation, infection, symptoms and NbBI-1 knockout reduces resistance to drought and cold stresses. **(A)** NbBI-1 promoted PVY accumulationin *N. benthamiana*. The relative RNA level of PVY was detected by qRT-PCR in PVY-infected plants. GAPDH was used as an internal reference. The relative accumulation of PVY in the leaves of plants was detected by ELISA. Leaves were collected 14 days after PVY infection. **(B)** NbBI-1 decreased resistance to PVY infection. After 14 days, WT, OK, and KD infected with PVY were selected to observe symptoms and to count the infection rate. **(C)** NbBI-1 promoted the pathogenicity of PVY. After 14 days, WT, OK, and KD infected with PVY were selected to observe and photograph. Row 1, bar, 1cm;Row 2, bar, 3 cm. **(D)** NbBI-1 knockout reduced resistance to drought. Take photos for the record after 10 days of stopping watering (Row 1, 4-week-old plants, bar, 2 cm; Row 2, 2-week-old plants). **(E)** NbBI-1 knockout reduced resistance to cold. Take photos for the record after 2 months of culture at 10 °C (4-week-old plants, bar, 2 cm). Data are mean ± SEM, asterisks indicate a significant difference (Student’s t-test, test, **: P<0.01).

To verify the role of NbBI-1 in the accumulation of PVY, we detected the relative accumulation of the virus in systematic leaves of WT, KD, and KO 14 days after PVY infection by qRT-PCR. Compared with WT (14 dpi), the accumulation of the PVY in KD (14 dpi) and KO (14 dpi) was markedly decreased (**Figure 3A**), the relative accumulations of PVY in WT (14 dpi), KD (14 dpi), and KO (14 dpi) were also detected by ELISA. The results were almost consistent with that of the qRT-PCR (**Figure 3A**). In the process of detecting virus titer was found that the rate of PVY infection in KD and KO plants was lower than that in WT plants (**Figure 3B**). The symptoms of leaf rolling in KO and KD were far less than in WT plants (**Figure 3C**). These results indicate that BI-1 had become a favorable tool for PVY infection and accumulation. However, the deletion of NbBI-1 reduced the tolerance of *N. benthamiana* to drought and microtherm. Compared with WT, KD, and KO showed significant vulnerability under drought stress (**Figure 3D**). And at the conditions of low temperatures, KO was the worst growth compared to WT and KD (**Figure 3E**). These experimental results suggest that although NbBI-1 utilized by PVY is not conducive to the resistance of *N. benthamiana* to PVY infection, NbBI-1 promotes resistance to abiotic stresses of drought and cold.

### 3.4 Transcriptome analysis role of NbBI-1 in PVY infection

Our results shows that NbBI-1 knockout inhibits PVY infection. The transcriptome sequencing was carried out on systematic leaf samples from WT (Control), WT (Treat), KO (Control), and KO (Treat) to further understand the role of NbBI-1 in PVY infection. It analyzed the differentially expressed genes (DEGs) in PVY infection of the WT and the KO.

**Figure 4:**
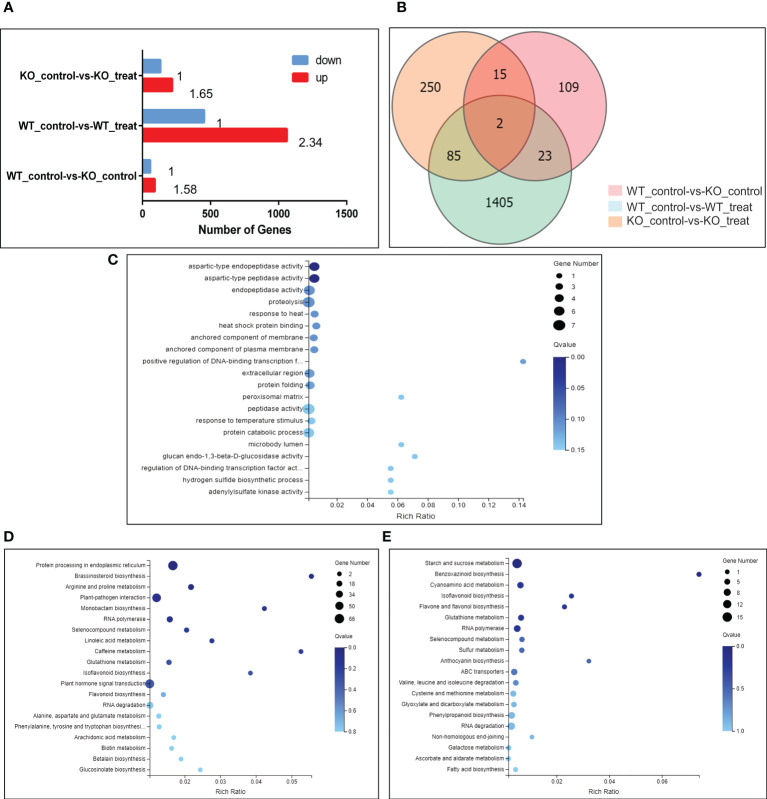
Changes in gene expression profiles of *N. benthamiana* (WT and KO) in response to PVY infection. **(A)** Several DEGs in pairwise comparisons of the 2 treatments. Qvalue (Adjusted Pvalue) <= 0.05. **(B)** Venn diagram showing the number of DEGs for different treatments. Qvalue (Adjusted Pvalue) <= 0.05. **(C)** Go enrichment map showing concentrations of DEG numbers of 85 genes that co-express differentially between the 2 treatments. Qvalue (Adjusted Pvalue) <= 0.05. **(D)** KEGG enrichment map showing concentrations of DEGs of WT_control-vs-WT_treat group. Qvalue (Adjusted Pvalue) <= 0.05. **(E)** KEGG enrichment map showing concentrations of DEGs of KO_control-vs-KO_treat group. Qvalue (Adjusted Pvalue) <= 0.05.

The results showed that a total of 1515 DEGs in the WT_control-vs-WT_treat group, among which 1061 genes were significantly up-regulated and 454 genes were significantly down-regulated, the number of up-regulated genes is 2.34 times rather than that of down-regulated genes. In the KO_control-vs-KO_treat group, a total of 352 DEGs of which 219 genes were significantly up-regulated and 133 genes were significantly down-regulated, the number of up-regulated genes is 1.65 times rather than that of down-regulated genes. A total of 149 DEGs were significantly expressed in the WT_control-vs-KO_control group, among which 91 genes were significantly up-regulated and 58 genes were significantly down-regulated, the number of up-regulated genes is 1.58 times greater than that of down-regulated genes (**Figure 4A**).

Using expressed gene data from multiple comparison groups, the Venn diagram was used to illustrate the number of genes among various comparison groups. 85 DEGs were discovered in the WT_control-vs-WT_treat group and the KO_control-vs-KO_treat group (**Figure 4B**). Clustering heat map analysis was performed on these 85 DEGs to understand the role of NbBI-1 in PVY-infection-inducing gene expression. Compared with the WT_control-vs-WT_treat group, changes in the amplitude of DEGs in the KO_control-vs-KO_treat group were significantly decreased (**Figure S3**; **Table S2**). The results showed that the NbBI-1 greatly increased the number of DEGs, enhanced changes’ amplitude of DEGs, and raised the proportion of up-regulated expression of DEGs in PVY-infected *N. benthamiana*. Additionally, GO enrichment analysis was performed on these 85 DEGs, and the results revealed that most DEGs were engaged in proteolysis (**Figure 4C**).

After analyzing the KEGG enrichment of the WT_control-vs-WT_treat group and KO_control-vs-KO_treat group, it was found that the DEGs of the 2 groups were involved in pathways including brassinosteroid biosynthesis, RNA polymerase, selenocompound metabolism, glutathione metabolism, isoflavonoid biosynthesis and RNA degradation (**Figures 4D, E**). And was also found three NbBI-1 mediated genes (enhancer of mRNA-decapping protein, pentatricopeptide repeat-containing protein, and anthocyanin) related to virus infection (**Table S3**). The capping mechanism of viral mRNA is an immune mechanism conducive to avoiding the host. Pokeweed antiviral protein (PAP) is a ribosome-inactivating protein, binds the cap structure and degrades RNA templates, such as Human immunodeficiency virus-1 RNA and Brome mosaic virus RNAs (Wang and Hudak, 2006). The turnip crinkle carmovirus (TCV) genomic RNA contains 5’ UTR and 3’ UTR. When viral mRNAs contain both 5’ and 3’ UTRs, TCV achieve optimal translational efficiency (Qu and Morris, 2000). A saliva-specific protein, named Aedes aegypti venom allergen-1 (AaVA-1), promotes dengue and Zika virus transmission by interacting with pentatricopeptide repeat-containing protein (PPRCP) to activate autophagy (Sun et al., 2020). The anthocyanins present in the Purple Majesty have a variety of anti-oxidative activities, anti-influenza virus activity, and anti-stomach cancer activity (Romano et al., 2022). Anthocyanin might play significant roles in plant defense against alfalfa mosaic virus (AMV) infection (Abdelkhalek et al., 2020). Enhancer of the mRNA-decapping protein gene, PPRCP gene, and anthocyanin gene, all responded to PVY infection mediated by NbBI-1 in *N. benthamiana*.

In animal cells, the BI-1 gene can interact with IRE1α and regulate the function of the IRE1α gene (Lisbona et al., 2009; Bailly-Maitre et al., 2010). The IRE1-bZIP60 pathway is involved in PVY infection (Gaguancela et al., 2016; Herath et al., 2020). IRE1α or IRE1b mediates the degradation of mRNA which may interfere with the induction of autophagy (Bao et al., 2018; Le Thomas et al., 2021), thus these mRNAs were also picked out to analyze. These genes decreased expression amplitude in the KO_control-vs-KO_treat group compared to the WT_control-vs-WT_treat group (**Table 1**). However, mRNA level of IRE1α was increased in the KO_control-vs-KO_treat group (**Table 1**). These might be the factor causing the relative decrease of mRNAs of some ATGs in PVY-infected plants via BI-1. Thus, these findings demonstrated that NbBI-1 gene deletion may increase *N. benthamiana* tolerance to PVY infection and NbBI-1 gene might regulate the alterations of mRNA of ATGs via IRE1α.

**Table 1:** RNAs encoding proteins that interfere with the induction of autophagy, and the IRE1 RNA.

Gene ID	log_2_(WT_treat/WT_control)	log_2_(KO_treat/KO_control)	NCBI	Description
Nbv5.1tr6356874	3.04	0.85	XP_009757217	suberization-associated anionic peroxidase-like
Nbv5.1tr6408909	2.90	1.77	NP_001312671	peroxidase N1-like precursor
Nbv5.1tr6426022	2.53	-0.12	XP_009600643	peroxidase 16-like
Nbv5.1tr6368425	2.35	1.46	XP_009771827	peroxidase 72-like
Nbv5.1tr6280238	2.23	0.72	PHT38375	L-ascorbate peroxidase 2, cytosolic
Nbv5.1tr6296835	1.16	0.72	XP_016492472	probable glutathione peroxidase 5
Nbv5.1tr6401022	1.12	0.66	XP_019228866	probable glutathione peroxidase 2
Nbv5.1tr6313181	5.80	1.50	XP_019230966	basic endochitinase
Nbv5.1tr6392363	2.56	2.20	OIT31482	chitinase 2
Nbv5.1tr6306030	2.34	0.93	XP_009770177	chitinase-3-like protein 1
Nbv5.1tr6368310	3.03	1.06	XP_009780913	glucan endo-1,3-beta-glucosidase, acidic isoform GI9-like
Nbv5.1tr6341649	2.81	0.50	XP_009786880	glucan endo-1,3-beta-glucosidase 5-like
Nbv5.1tr6280022	2.49	0.80	OIT40131	glucan endo-1,3-beta-glucosidase, acidic isoform gi9
Nbv5.1tr6424171	1.51	0.85	XP_009758632	glucan endo-1,3-beta-glucosidase 7-like isoform X1
Nbv5.1tr6399677	0.83	0.27	XP_009789910	glucan endo-1,3-beta-glucosidase-like
Nbv5.1tr6304235	1.42	0.76	XP_022732899	endoribonuclease Dicer homolog 2-like isoform X5
Nbv5.1tr6294664	1.13	0.58	XP_019231893	3’-5’ exoribonuclease 1-like
Nbv5.1tr6371605	1.69	0.75	AAT45202	lipid transfer protein 1 precursor, partial
Nbv5.1tr6423902	0.07	1.81	XP_009791965	endoribonuclease IRE1α

### 3.5 NbBI-1 mediates the degradation of NIb by NbATG6

**Figure 5:**
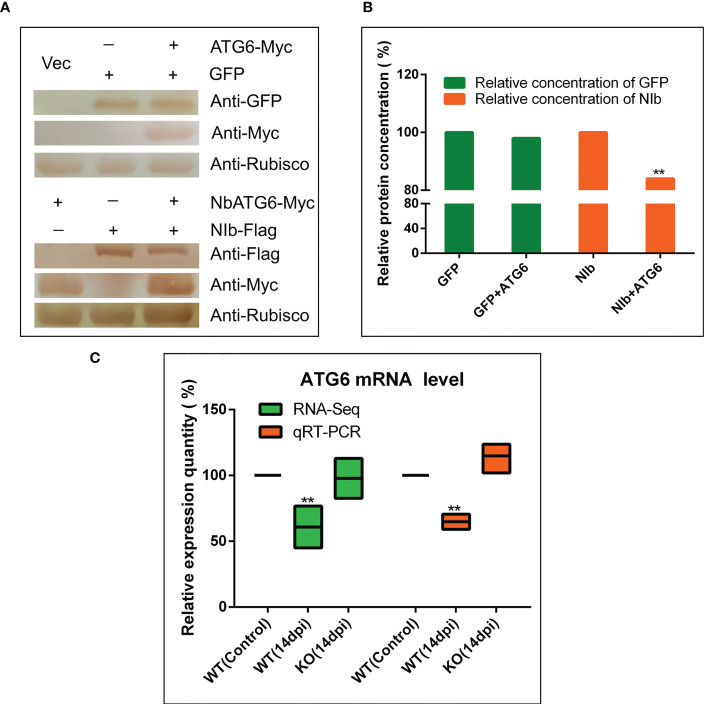
NbBI-1 mediated the degradation of NIb by NbATG6. **(A, B)** NbATG6 degraded PVY NIb. NbATG6, NIb, and GFP were detected by West blot after they co-expressed in plant leaves for 3 days. The western blot detected results were transposed gray and calculated the gray value of these protein bands by photoshop 2020. **(C)** NbBI-1 mediated the decrease in the expression of NbATG6 caused by PVY infection (qRT-PCR and RNA-Seq). Leaves were collected at 14 dpi. (Student’st-test, test, **: P<0.01).

It has been shown that NbBeclin1 (ATG6) mediates the degradation of NIb of TuMV (Li et al., 2018). Thus, the plasmids including PVY NIb with Myc protein tag and NbATG6 with Flag protein tag were constructed to investigate whether NbATG6 could also degrade PVY NIb. It was tested the relative content of NbATG6 and NIb by western blot after transiently co-express NbATG6 and NIb in leaves of *N. benthamiana*. As a result, the protein concentration of NIb decreased significantly (**Figures 5A, B**), suggesting a degrading effect of NbATG6 for NIb.

To confirm the effect of NbBI-1 knockout on the mRNA expression level of the NbATG6, qRT-PCR, and RNA-seq were performed. The results showed that NbATG6 mRNA was significantly down-regulated in PVY-infected WT (14 dpi), relative to PVY-free control (**Figure 5C**). However, there were no obvious changes in the mRNA level of NbATG6 in PVY-infected KO (14 dpi) (**Figure 5C**). Based on the above results, NbBI-1 might mediate the degradation of PVY NIb by NbATG6 in PVY infection.

## 4 Discussion

Autophagy has many biological functions in plants, and it is closely related to plant growth, development, flowering, and fruiting (Cheng et al., 2022). It will also cause autophagy when exposed to exogenous biotic or abiotic stress. Various pathogens, including fungi, bacteria, and viruses, have the potential to cause autophagy. The difference is that viral proteins that trigger autophagy are mostly produced in the host cell’s endoplasmic reticulum; viral proteins could lead to endoplasmic reticulum stress (Verchot, 2016; Yang et al., 2016), and autophagy may be triggered through the UPR pathway (Li et al., 2020; Verchot and Pajerowska-Mukhtar, 2021); resulting in the degradation of viral proteins (Li et al., 2018; Jiang et al., 2020). Viral proteins produced in host cells can interact with the endoplasmic reticulum in an extensive and complex manner (Zhang and Wang, 2012; Gayral et al., 2020; Herath et al., 2020; Ali et al., 2022). In the process of co-evolution of viruses and hosts, viruses may gain the ability to regulate autophagy and exist in a way, conducive to viral replication (Kushwaha et al., 2019; Ismayil et al., 2020). However, the research in this area has lagged behind the antiviral autophagy mechanism.

This work proves that PVY P3/P3N-PIPO interacted with NbBI-1, and the mutant that eliminated the NbBI-1 gene, considerably reduced virus accumulation and symptom severity. It has been confirmed that BI-1 interacts with ATG6 (Xu et al., 2017), and ATG6, VPS15, and VPS34 form the phosphatidylinositol 3-kinase (PI3K) complex (Mizushima et al., 2011). In this study, the expression level of NbATG6 was down-regulated under PVY infection. However, in the BI-1 knockout mutant, the expression level of NbATG6 did not change significantly after PVY infection. This contrasts with prior research results that showed ATG6 expressed more strongly to resist RSV infection Jiang et al., 2020). But, our results are similar to that of silencing or knockdown of ATG6-promoting TuMV infection in *N. benthamiana* or Arabidopsis plants (Li et al., 2018). Since it has been reported that ATG6 can degrade TuMV NIb (Li et al., 2018). The evidence was present in this research that NbATG6 can also degrade PVY NIb in *N. benthamiana*. The data suggested that in PVY infection, the NbBI-1 repressed the expression of the NbATG6 gene, thereby reducing the degradation of viral protein NIb and enhancing PVY replication. Conversely, eliminating the NbBI-1 gene relieves the inhibitory on NbATG6 gene expression and reduced PVY replication. It would be interesting to determine how BI-1 regulates the expression of the ATG6 gene during PVY infection.

To explore the role of the NbBI-1 gene on the effect of PVY infections, the transcriptome data from PVY-infected wild-type *N. benthamiana* were compared with those from NbBI-1 knockout mutant. It was found that in PVY-infected NbBI-1 knockout mutants, the number and magnitude of changes in gene expression showed an overall significant decrease, including the genes involved in proteolysis enzymes. Coxsackievirus B5 (CVB5) infection induced ubiquitin-mediated proteolysis and signaling pathways (Li et al., 2022b). The NIa protease of PPV or TuMV proteases cleaves host proteins by proteolysis to facilitate virus infection (Xiao et al., 2022). According to the research of Chenopodium quinoa mitovirus 1 (CqMV1), there is up-regulation of functional modules involved in amino acid catabolism, proteolysis, folding/stress response, and redox homeostasis in quinoa-infected plants (Di Silvestre et al., 2022). These results indicated that complete elimination of the NbBI-1 gene inhibits the changes in PVY-infected-related genes and enhances *N. benthamiana* tolerance to PVY infection. Recent studies have shown that IRE1 degrades mRNAs encoding proteins that interfere with the induction of autophagy by ER stress in Arabidopsis thaliana (Bao et al., 2018). In a human cell, IRE1 helps to mitigate ER stress by cleaving specific and nonspecific mRNAs via regulated IRE1-dependent decay (RIDD) and RIDD lacking endo-motif (RIDDLE) respectively (Le Thomas et al., 2021). Therefore, it suggested that NbBI-1 might inhibit the expression of the related autophagy gene ATG6 via the IRE1-mediated RIDD pathway during PVY infection.

BI-1 is a multifunctional protein that is also widely distributed in various tissues and cells (Lebeaupin et al., 2020). The deletion and reduction of BI-1 may seriously impact *N. benthamiana*. The complete elimination of NbBI-1 promoted the growth and development of *N. benthamiana*. Our experimental results show that NbBI-1 negatively regulates the growth and development of *N. benthamiana*. This is similar to the results of research that BI-1 negatively regulates the growth and development of fungal hyphae (Xie et al., 2019). However, it has also been reported that BI-1 does not affect normal growth and development. BI-1-deficient mice can survive and develop normally (Chae et al., 2004). And there is no difference between BI-1 deficiency Arabidopsis and wild-type Arabidopsis in normal growth (Watanabe and Lam, 2006). Overexpression of BI-1 plants was fertile and did not display obvious developmental alterations compared to wild-type parents (Babaeizad et al., 2009). These seem to indicate that BI-1 has different functions for growth and development in different species

It is important to note that the BI-1 gene has opposite functions: inhibiting autophagy and promoting autophagy. In BI-1-deficient mice, the autophagy activity of liver and kidney cells is enhanced, which may be dependent on the IRE1/UPR pathway (Castillo et al., 2011). Autophagy is more likely to occur in *N. tabacum* cell lines where the BI-1 gene is antisense downregulated when exposed to sucrose starvation (Bolduc and Brisson, 2002). These show that BI-1 inhibits autophagy. However, another study showed that BI-1 promotes autophagy in an IP3R-dependent manner and BI-1-deficient mice showed attenuated basal autophagy levels (Sano et al., 2012). In plants, silencing of the BI-1 in *N. benthamiana* reduced the autophagic activity induced by TMV and MV (Xu et al., 2017). These show that BI-1 promotes autophagy.

When agroinfiltration is used to co-deliver PVY-GFP with BI-1 silencing constructs to *N. benthamiana* leaves compared with the control leaves, systemic accumulation of PVY-GFP increased significantly (Gaguancela et al., 2016). As far as PVY are concerned, as one of the most important pathogens, in the same host, PVY can produce asymptomatic, mild symptoms, and even necrosis (HR) depending on different virus strains (Tamisier et al., 2022). In this study, a PVY strain that caused mild symptoms, and no necrotic symptoms was utilized. Similarly, a PVY strain may cause various symptoms in various hosts depending on their resistance level (Baebler et al., 2020).

## 5 Conclusion

**Figure 6:**
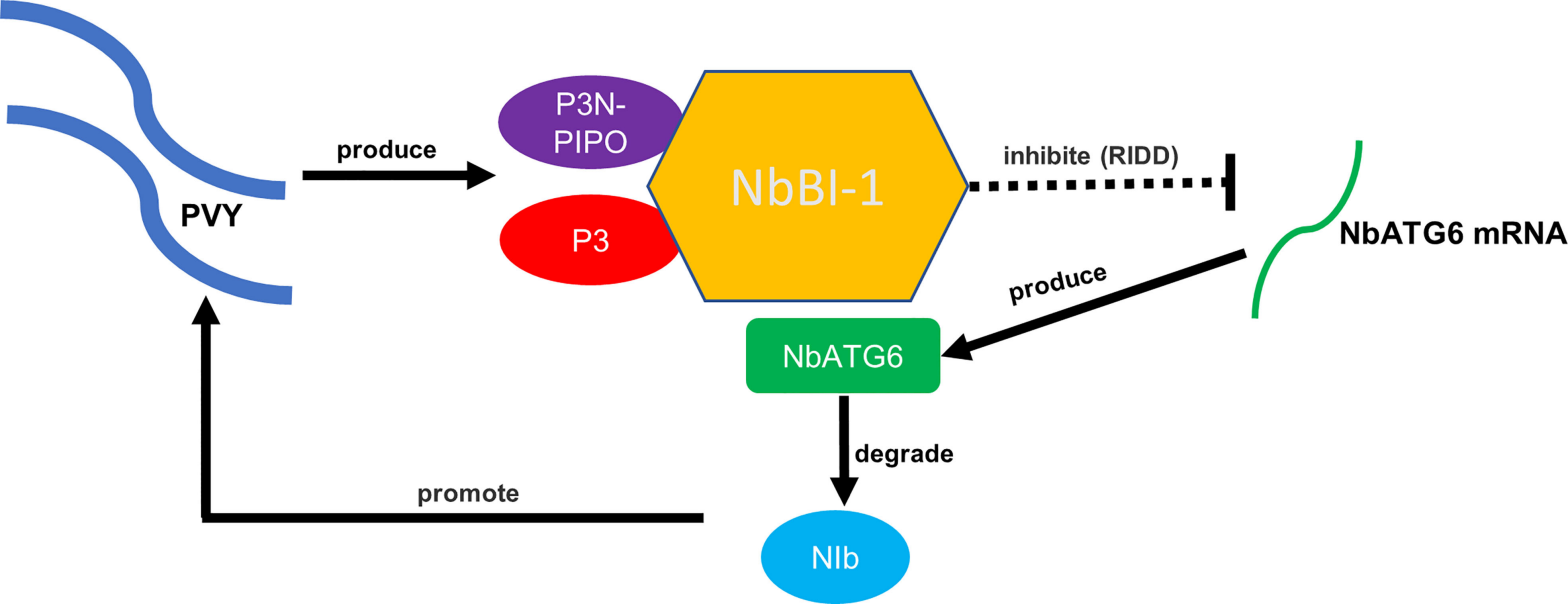
A working model of NbBI-1-mediated PVY infection breaking through the autophagic defense of *N. benthamiana*.

This research work concluded with a model to summarize how the virus hijacks BI-1-mediated regulation of autophagy to promote viral replication (**Figure 6**). In the case of PVY infection, viral P3 and P3N-PIPO interact with NbBI-1. The deletion of NbBI-1 enhanced the growth and development of tobacco and reduced the resistance to abiotic stresses (drought and low temperature). In addition, the deletion of NbBI-1 enhance the resistance to PVY infection and weakened the gene expression regulation induced by PVY infection. NbBI-1 restricts the production of NbATG6 genes in the autophagy-related genes likely through the RIDD pathway, which is regulated by IRE1 according to the analysis of transcriptome data. This would prevent NbATG6 from degrading PVY RNA-dependent RNA polymerase NIb, promoting viral replication.

### 5.1 Future perspective

As an autophagy-related protein, ATG6 participates in the regulation of autophagy in plants. Whether the interaction between P3/P3N-PIPO and BI-1 is involved in the autophagy induced by PVY infection in *N. benthamiana*. How does NbATG6 degrade PVY NIb? And how does NbBI-1 reduce NbATG6 mRNA levels through the RIDD pathway? These may be the focus of our subsequent research.

## Data availability statement

The original contributions presented in the study are included in the article/**Supplementary Materials**, further inquiries can be directed to the corresponding author/s.

## Author contributions

ZQ and RW: conceptualization and writing-original draft preparation. ZQ: methodology and formal analysis. LZ, QL, YC, and ZQ: investigation. RW and BC: resources and supervision. ZQ and RW: data curation. ZQ, SA, and RW: writing-review and editing. All authors contributed to the article and approved the submitted version.
