# Supplementary material for: P3/P3N-PIPO of PVY interacting with BI-1 inhibits the degradation of NIb by ATG6 to facilitate virus replication in N. benthamiana
Source: Front Plant Sci. 2023 Apr 17;14:1183144. doi: 10.3389/fpls.2023.1183144 (PMC10149851; doi:10.3389/fpls.2023.1183144)
Supplement: Supplementary file 1 [file DataSheet_1.pdf]

A

Gene name	NbBI-1
Intermediate carrier skeleton	PBSK-RTM
Forward fragment amplification primers	BI-F2: cgggatccatcgctggagttacgattc
	BI-R2: gctctagacagtgcagctgccatcagaa
Reverse fragment amplification primers	BI-F3: cgagctcatcgctggagttacgattc
	BI-R3: atttgcggccgccagtgcagctgccatcagaa
Interference carrier skeleton	pCAMBIA2301-ky

B

NbBI-1
atcgctggagttacgattctcttaagaact
tccgccagatctctccctttgttcaaactc
atctcaaaaaggtctacctttcattatgct
gtgctttaattgcttcggctgctggagctt
accttcacattctttggaacatcggtggtt
tacttacgacgctgggatgcgtgggaag
catagtatggctgatggcgactcctctgt
atgaagagcaaaagaggatagcacttct
gatggcagctgcactg

RTM
acgttgtaagtctatttttgactcttcttttt
ctccgtcacaatttctacttccaactaaaa
tgctaagaacatggttataactttttttttat
aacttaatatgtgatttggacccagcaga
taga

C

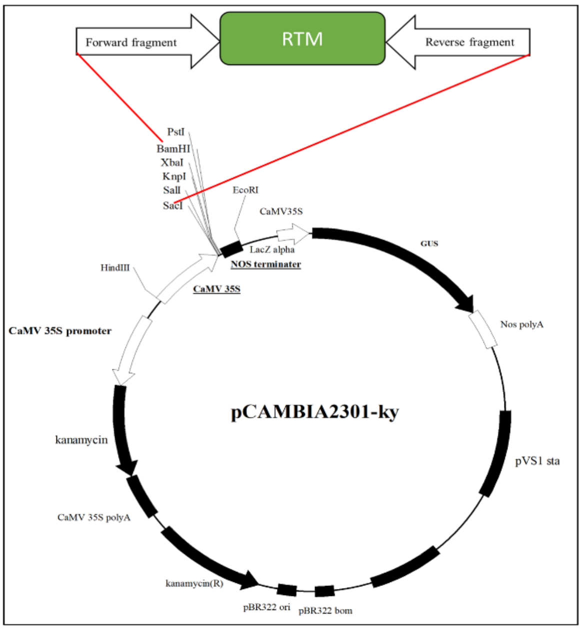

Figure supplementary 1. KD obtained by RNA interference technology. (**A)** Information on infection vector of NbBI-1 gene of *N. benthamiana*. (**B**) NbBI-1 Interfering Fragment & RTM Fragment. (**C)** Interfering vector construction strategy.

A

**Gene name**	NbBI-1
**CRISPR/Cas9 vectors**	PKSE401
**gRNA target sequence**	gaacaaagggagagatctggcgg
**Target sequence primers**	BIgRNA-F: attggaacaaagggagagatctggcgg
	BIgRNA-R: aaacccgccagatctctccctttgttc

B

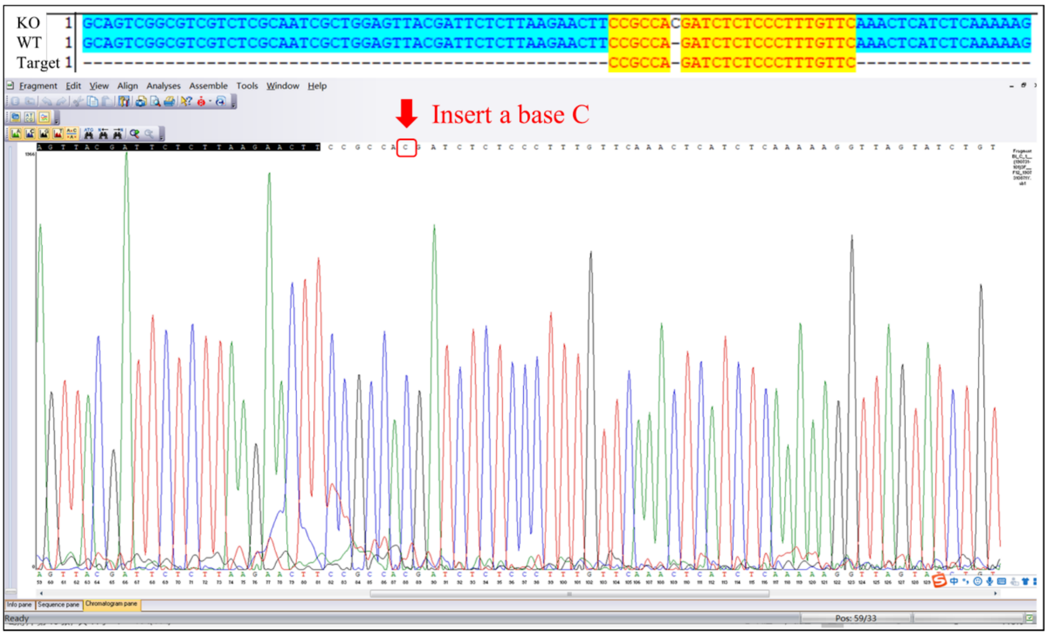

Figure supplementary 2. KO obtained by CRISPR/Cas9. (**A**) Information on gene editing vector and target. (**B**) Test result of Knockout of *NbBI-1* gene

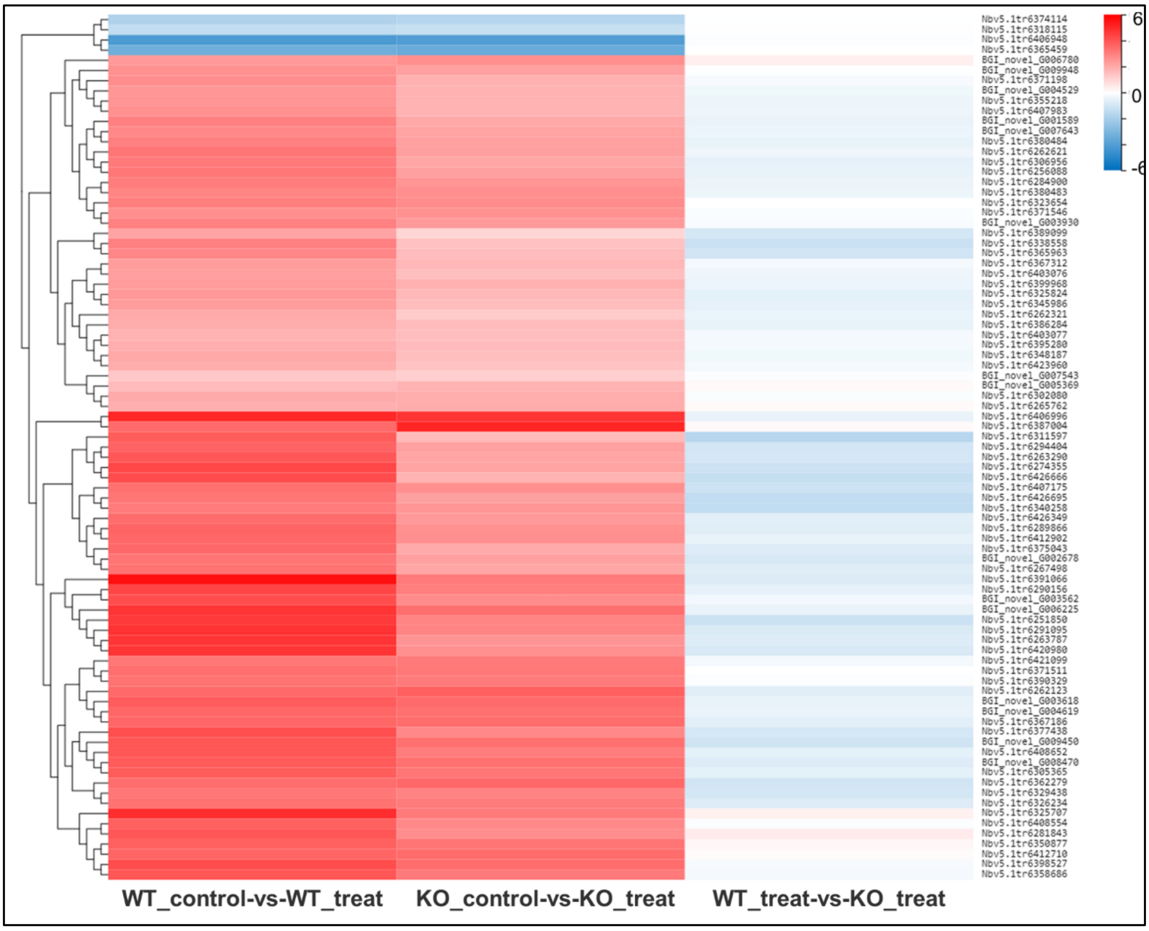

Figure supplementary 3. Clustering heat map analysis showing relative expression levels of DEG numbers of 85 genes that co-express differentially between the 2 treatments. Qvalue (Adjusted Pvalue) <= 0.05.

Table supplementary 1. The oligonucleotide primer set used for qRT-PCR

Goal Name	Fwd Primer (5'-3')	Rev Primer (5'-3')	Annotation
GAPDH	ggctgaagctggtgctgatt	ggtagtgcaactggcattgg	JQ256517.1
NbBI-1	ttaagaacttccgccagat	cgccatcagccatactat	XM_019380726.1
PVY	gacgcagaagcagaggcattc	tcacagtccacgataccaacatcta	PVY^{N-Wi}
NbATG6	aggagttggaggagcggta	gcatcattcagcacattagtcttc	AY701316.1

Table supplementary 2. Relative expression levels of DEG numbers of 85 genes that co-express differentially between the 2 treatments. Qvalue (Adjusted Pvalue) <= 0.05.

No.	Gene ID	log_2(WT_treat/WT_control)	log_2(KO_treat/KO_control)	NCBI/Description
1	BGI_novel_G001589	3	2.03	
2	BGI_novel_G002678	3.27	2.23	
3	BGI_novel_G003562	4.18	2.7	
4	BGI_novel_G003618	3.81	3.48	
5	BGI_novel_G003930	2.97	2.37	
6	BGI_novel_G004529	2.45	1.81	
7	BGI_novel_G004619	3.56	3.36	
8	BGI_novel_G005369	1.59	1.78	
9	BGI_novel_G006225	4.74	3.4	
10	BGI_novel_G006780	2.38	2.65	
11	BGI_novel_G007543	1.28	1.15	
12	BGI_novel_G007643	2.81	2.16	
13	BGI_novel_G008470	3.85	3.33	
14	BGI_novel_G009450	3.97	3.33	

15	BGI_novel_G009948	2.58	2.2	
16	Nbv5.1tr6251850	4.65	2.86	XP_019255718.1/cucumber peeling cupredoxin-like
17	Nbv5.1tr6256088	3.2	2.24	XP_019245555.1/uncharacterized protein LOC109225373
18	Nbv5.1tr6262123	3.52	3.72	XP_009780913.1/glucan endo-1,3-beta-glucosidase, acidic isoform GI9-like
19	Nbv5.1tr6262321	2	1.22	XP_009802815.1/F-box/kelch-repeat protein At1g15670-like
20	Nbv5.1tr6262621	3.23	2.24	XP_019233692.1/pleiotropic drug resistance protein 1
21	Nbv5.1tr6263290	3.96	2.11	XP_016436463.1/WRKY transcription factor 70
22	Nbv5.1tr6263787	4.73	2.52	XP_016490011.1/adenylyl-sulfate kinase 3-like
23	Nbv5.1tr6265762	1.88	1.96	XP_009802815.1/F-box/kelch-repeat protein At1g15670-like
24	Nbv5.1tr6267498	3.29	2.11	XP_019249046.1/uncharacterized protein LOC109228351
25	Nbv5.1tr6274355	4.32	2.16	XP_009760864.1/probable WRKY transcription factor 70
26	Nbv5.1tr6281843	3.96	2.67	XP_019232872.1/protein EXORDIUM-like
27	Nbv5.1tr6284900	3.05	2.5	XP_019223933.1/ vacuolar inhibitor of fructosidase 1-like
28	Nbv5.1tr6289866	3.64	2.58	XP_011079327.1/probable alpha,alpha-trehalose-phosphate synthase
29	Nbv5.1tr6290156	4.35	3.02	XP_009629719.1/aspartyl protease family protein At5g10770-like

30	Nbv5.1tr6291095	4.79	2.88	XP_019258468.1/GDSL esterase/lipase 1-like isoform X2
31	Nbv5.1tr6294404	3.66	2.21	XP_009792121.1/F-box protein At5g03970-like
32	Nbv5.1tr6302080	1.98	1.93	XP_009802815.1|F-box/kelch-repeat protein At1g15670-like
33	Nbv5.1tr6305365	3.83	3.23	XP_019263809.1/nicotinamidase 1-like isoform X1
34	Nbv5.1tr6306956	3.13	2.1	XP_009778648.1/pentatricopeptide repeat-containing protein At5g27460
35	Nbv5.1tr6311597	3.83	1.63	XP_009792440.1/chaperone protein ClpB1-like
36	Nbv5.1tr6318115	-1.44	-1.34	XP_019241291.1|UDP-glycosyltransferase 74E2-like
37	Nbv5.1tr6323654	3.02	2.72	XP_009801873.1/expansin-like B1
38	Nbv5.1tr6325707	4.91	3.12	XP_009598446.1/protein CELLULOSE SYNTHASE INTERACTIVE 3
39	Nbv5.1tr6325824	2.43	1.64	XP_019254472.1/NAC domain-containing protein 14-like
40	Nbv5.1tr6326234	3.3	3.08	XP_009789603.1/outer envelope pore protein 16-2, chloroplastic
41	Nbv5.1tr6329438	3.2	2.92	XP_009768610.1/calcium-binding protein CML41
42	Nbv5.1tr6338558	2.97	1.43	AGY48887.1/MIP1.4b
43	Nbv5.1tr6340258	3.08	2.45	XP_019251689.1/early nodulin-like protein 2
44	Nbv5.1tr6345986	2.31	1.55	XP_009763590.1/apolipoprotein D-like
45	Nbv5.1tr6348187	2.02	1.52	XP_009802815.1/ kelch-repeat protein At1g15670-like

46	Nbv5.1tr6350877	3.74	3.26	AGW21710.1/auxin repressed protein 1, partial
47	Nbv5.1tr6355218	2.49	1.78	XP_019243705.1/aspartyl protease family protein At5g10770-like
48	Nbv5.1tr6358686	3.98	3.14	XP_009766613.1/receptor-like protein kinase At5g24010
49	Nbv5.1tr6362279	3.4	3.54	XP_009790678.1/glutathione transferase GST 23-like
50	Nbv5.1tr6365459	-3.36	-3.53	XP_019224985.1/FHA domain-containing protein PS1-like
51	Nbv5.1tr6365963	2.82	1.57	AGY48887.1/MIP1.4b
52	Nbv5.1tr6367186	3.57	3.44	XP_019234660.1/14 kDa proline-rich protein DC2.15-like
53	Nbv5.1tr6367312	2.33	1.68	XP_009791526.1/uncharacterized protein LOC104238762
54	Nbv5.1tr6371198	2.68	1.86	XP_009787077.1|glycine-rich cell wall structural protein 1.8-like
55	Nbv5.1tr6371511	3.39	3.15	XP_019228658.1/peptidyl-prolyl cis-trans isomerase FKBP62-like
56	Nbv5.1tr6371546	2.67	2.58	XP_015079103.1/sugar transport protein 8-like
57	Nbv5.1tr6374114	-1.86	-1.73	XP_009782739.1/nucleolar protein 56-like
58	Nbv5.1tr6375043	3.56	2	XP_016441917.1/carotenoid cleavage dioxygenase 4, chloroplastic
59	Nbv5.1tr6377438	4.13	2.77	KYP66812.1/Retrovirus-related Pol polyprotein from transposon TNT 1-94
60	Nbv5.1tr6380483	2.9	2.63	OIT04930.1/aspartyl protease family protein
61	Nbv5.1tr6380484	2.98	2.21	OIT04930.1/aspartyl protease family protein

62	Nbv5.1tr6386284	1.95	1.59	XP_019232958.1/U-box domain-containing protein 38-like
63	Nbv5.1tr6387004	3.49	5.1	XP_009799719.1/strictosidine synthase 1-like
64	Nbv5.1tr6389099	2.14	0.97	AGY48887.1/MIP1.4b
65	Nbv5.1tr6390329	3.3	3.14	XP_016486674.1/zinc finger Ran-binding domain-containing protein 2-like
66	Nbv5.1tr6391066	5.61	3.1	XP_009803076.1/4-coumarate--CoA ligase-like 10
67	Nbv5.1tr6395280	1.88	1.59	XP_009802815.1/F-box/kelch-repeat protein At1g15670-like
68	Nbv5.1tr6398527	4.22	3.43	XP_009589046.1/extensin-2-like isoform X1
69	Nbv5.1tr6399968	2.31	1.87	XP_019254472.1/NAC domain-containing protein 14-like
70	Nbv5.1tr6403076	2.26	1.5	XP_019243705.1/aspartyl protease family protein At5g10770-like
71	Nbv5.1tr6403077	1.79	1.55	XP_009622884.1/uncharacterized protein LOC104114205 isoform X1
72	Nbv5.1tr6406948	-4.2	-4.07	XP_009800313.1/golgin candidate 2 isoform X1
73	Nbv5.1tr6406996	5.06	4.76	XP_009623569.1/probable mannitol dehydrogenase
74	Nbv5.1tr6407175	3.34	2.67	XP_009784386.1/uncharacterized protein LOC104232799
75	Nbv5.1tr6407983	2.58	1.77	XP_009762638.1/metal-nicotianamine transporter YSL1-like isoform X3
76	Nbv5.1tr6408554	3.74	2.77	XP_009782398.1/osmotin
77	Nbv5.1tr6408652	3.97	3.06	XP_009787582.1/uncharacterized protein LOC104235494

78	Nbv5.1tr6412710	3.62	3.45	XP_009788085.1/auxin-repressed 12.5 kDa protein-like
79	Nbv5.1tr6412902	3.57	2.62	ACA79924.1/chloroplast N receptor-interacting protein 1
80	Nbv5.1tr6420980	4.78	2.61	XP_019159582.1/sugar transport protein 13 isoform X1
81	Nbv5.1tr6421099	3.2	3.11	XP_019252157.1/pleiotropic drug resistance protein 1-like
82	Nbv5.1tr6423960	1.91	1.42	XP_009802815.1/F-box/kelch-repeat protein At1g15670-like
83	Nbv5.1tr6426349	3.46	2.4	XP_019245638.1/|protein DOWNY MILDEW RESISTANCE 6
84	Nbv5.1tr6426666	4.18	1.79	AGZ61934.1/mitochondrial alternative oxidase 1a
85	Nbv5.1tr6426695	3.23	2.23	XP_019256114.1/cytochrome P450 CYP72A219-like isoform X1

Table supplementary 3. Relative expression levels of DEGs that co-express differentially between the 2 treatments. Qvalue (Adjusted Pvalue) <= 0.05.

**Gene ID**	**$\log_2$(WT_treat /WT_control)**	**$\log_2$(KO_treat /KO_control)**	**NCBI/Description**
Nbv5.1tr6419219	1.72	3.58	XP_009780406/enhancer of mRNA-decapping protein
Nbv5.1tr6303956	3.13	2.10	XP_009778648/pentatricopeptide repeat-containning protein
Nbv5.1tr6297626	/	3.64	XP_009769443/anthocyanin
